# Radiocarbon Dating of Mortar Fragments from the Fresco of a Romanian Monastery: A Field Study

**DOI:** 10.3390/ma18051149

**Published:** 2025-03-04

**Authors:** Marioara Abrudeanu, Corina Anca Simion, Adriana Elena Valcea, Maria Valentina Ilie, Elena Alexandra Ispas, Maria Loredana Marin, Dragos Alexandru Mirea, Dan Cristian Olteanu, Cristian Manailescu, Alexandru Razvan Petre, Denis Aurelian Negrea, Sorin Georgian Moga, Izabela Maris, Dorin Grecu, Gheorghe Garbea, Flavio Nicolae Finta, Mircea Ionut Petrescu

**Affiliations:** 1 Doctoral School of Materials Science and Engineering, National University of Science and Technology POLITEHNICA Bucharest, 313 Splaiul Independenţei, Sector 6, 060042 Bucharest, Romania; adriana.valcea31@gmail.com (A.E.V.); fintaflavio@gmail.com (F.N.F.); 2 Technical Sciences Academy of Romania, 118 Calea Victoriei, Sector 1, 010093 Bucharest, Romania; 3 Horia Hulubei National Institute for R&D in Physics and Nuclear Engineering (IFIN-HH), 30 Reactorului St., P.O. Box–MG-06, 077125 Magurele, Romania; maria.ilie@nipne.ro (M.V.I.); elena_ispas_07@yahoo.com (E.A.I.); marinmarialoredana@yahoo.com (M.L.M.); dragos.mirea@nipne.ro (D.A.M.); cristian.olteanu89@yahoo.com (D.C.O.); cristian.manailescu@nipne.ro (C.M.); alexpetre@nipne.ro (A.R.P.); 4 Regional Center of Research & Development for Materials, Processes and Innovative Products Dedicated to the Automotive Industry (CRCD-AUTO), National University of Science and Technology POLITEHNICA Bucharest, Universiy Center Pitesti, 1 Targu din Vale St., 110040 Pitesti, Romania; aurelian.negrea@upb.ro (D.A.N.); sorin_georgian.moga@upb.ro (S.G.M.); 5 Department of Mineralogy, Faculty of Geology and Geophysics, University of Bucharest, 1 N. Bălcescu Ave., 011401 Bucharest, Romania; izabela@contentlogic.ro; 6 Faculty of Theology, Letters, History and Arts, National University of Science and Technology POLITEHNICA Bucharest, University Center Pitesti, 1 Targu din Vale St., 110040 Pitesti, Romania; dorin_grecuro@yahoo.com (D.G.); garbea_59@yahoo.com (G.G.); 7 Department of Engineering and Management of Metallic Materials Casting, Faculty of Materials Science and Engineering, National University of Science and Technology POLITEHNICA Bucharest, Splaiul Independenţei nr. 313, Sector 6, 060042 Bucharest, Romania; ionut.petrescu@upb.ro

**Keywords:** cave painting, radiocarbon dating, electron microscopy, compositional analysis, accelerator mass spectrometry

## Abstract

The stone Ensemble from Corbii de Piatrǎ Romania arouses a continuous scientific interest, with the final goal being to obtain an exhaustive and multidisciplinary package of results that will become the support of an extensive restoration project. The cave painting stands out as the most important and most affected by the advanced degradation among the historical monuments in Romania. This article provides for the first time a radiocarbon dating of the first forms of painting by establishing the age of the mortar/plaster used as a pictorial support. Being a very complex context from the point of view of the type of datable material and the disappearance over time through degradation of other elements that would ensure a simpler and more reliable radiocarbon dating (such as the straws used to form the material), it was necessary to use a multidisciplinary approach for the selection of samples and for supporting the radiocarbon results. The set of analyses consisted of visualization techniques through microscopy and compositional analysis, providing information on the similarities/differences between the samples, the degradation mechanisms/impurities and the quality of the calcium carbonate dated by the Accelerator Mass Spectrometry (AMS) technique. The results supported each other, ensured the selection of reliable radiocarbon data and established the most probable moment of the early interventions, namely the two phases corresponding to the 14th century.

## 1. Historic

Considered to be one of the oldest places of worship, used since the second century AD and sheltered from persecutions and barbarian raids, the current Corbii de Piatrǎ/Stone Crows cave monastery in Romania is built on an old Dacian site [1].

The first mention of the Corbii de Piatrǎ dates back to 15 April 1456, when Vladislav II, the Voivode of Wallachia, issued an act in Old Slavic language in Targoviste, confirming Mogos’ ownership of the villages: “All the Corbii, from Aghiş downward, and all the Corbii-de-Piatrǎ, and the pasture from Miceşti, and the mill waters, and half of Mǎlureni, because they are ancient and rightful pastures of Mogoş (…)” [2]. Corbii de Piatrǎ became the first nunnery on the territory of Romania, documented on 23 June 1512, when the nun Magdalina (Muşa in lay life), the hereditary heiress of the estate in Corbi, re-established the monastery, which acquired the status of a princely monastery, donating it to Voivode Neagoe Basarab [3].

**Figure 1:**
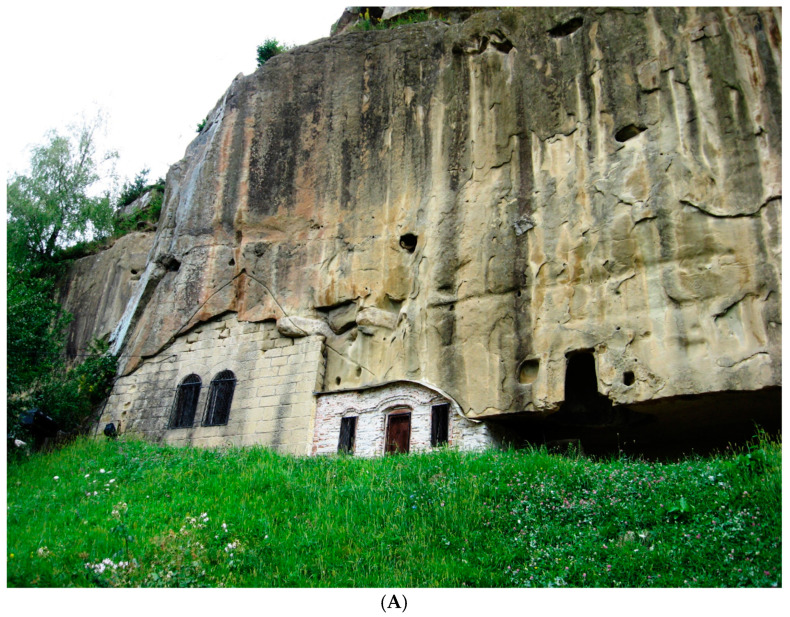

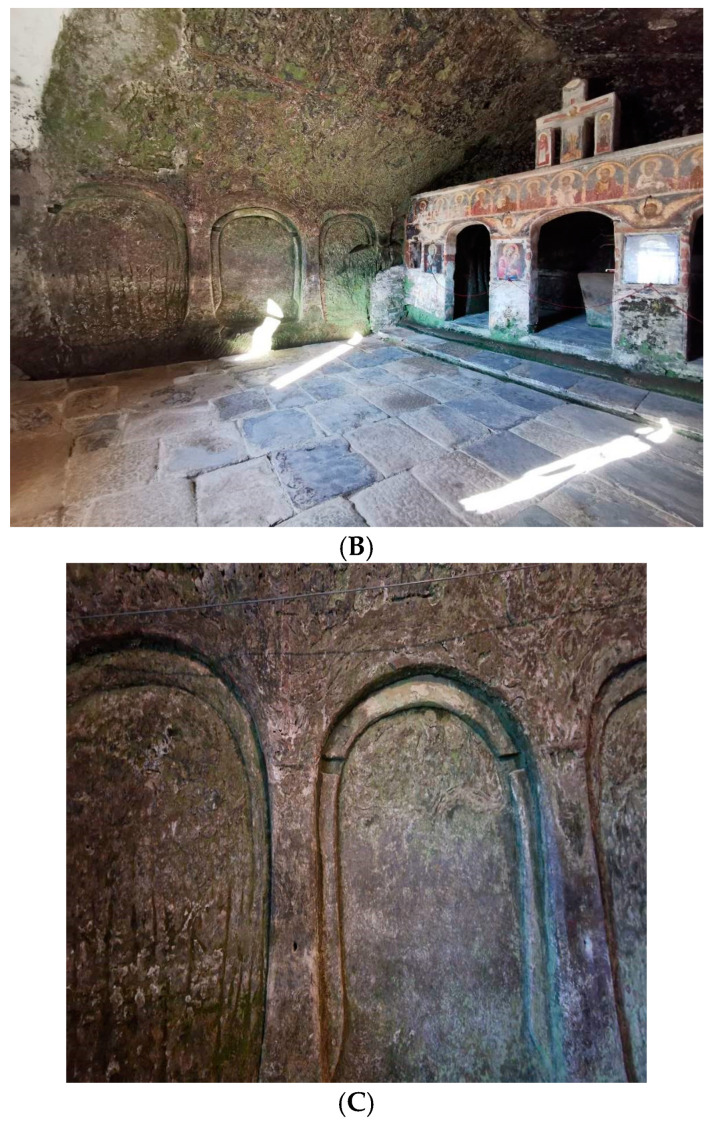
Corbii de Piatrǎ rupestral church: (**A**) exterior view, tiled wall (Photo: Simion, C. A.); (**B**,**C**) interior view for ship-type structure (Photo: Valcea, A. E.).

The architecture of the Corbii de Piatră cave church (Figure 1A) is typologically derived from hall-type churches, with two altars dedicated to a double rite, characteristic of the Byzantine world from the 10th century (Figure 1B,C) [1,2,3,4,5].

It is considered that the fresco painting, in a purely Byzantine style, was made at the beginning of the 14th century, during the reign of Voivode Basarab I the Founder (1310–1352), having the wall excavated in the sandstone (naturally and/or man-made) as support. Over time, water infiltrations through the sandstone wall, temperature variations and biodegradation processes determined the advanced deterioration of the frescoes.

The previous studies [6,7,8,9,10,11,12,13] carried out for the fresco from Corbii de Piatrǎ provide restorers with data on their execution materials. The recent characterization of two fragments of the church fresco, from areas with different behavior under similar conditions [6,7], posed the problem of their dating and their inclusion in the chronology of the fresco.

## 2. Geological, Mineralogical and Hydrogeological Considerations

**Figure 2:**
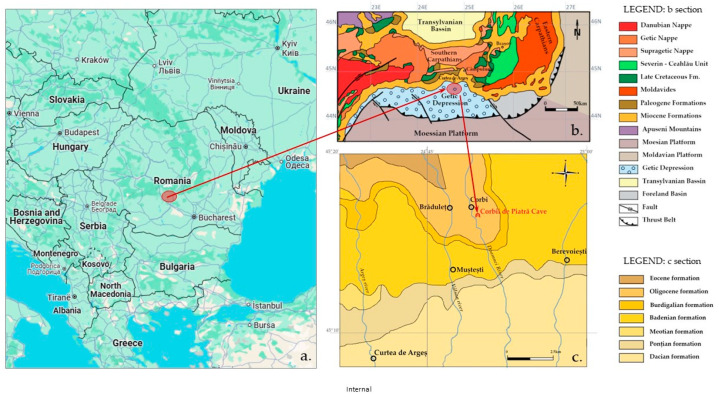
Map with the researched area, modified after Ghiran et al., 2023 [14]; (**a**) Occurrence of Southern Carpathian (Google Earth source); (**b**) Simplified tectonic map of the Getic Depression, modified from Sandulescu, 1984 [15]; (**c**) The studied area with location of the Corbii de Piatră monastery on Oligocene Corbi Formation, modified after Murgeanu et al., 1967 [16].

From a geological point of view, the site containing Corbii de Piatrǎ Monastery is located in the Getic Depression (Figure 2), a narrow sedimentary basin in the Southern Carpathian foreland with sediments from the Cretaceous to Miocene ages [14]. The monastery was excavated in massive sandstones of the Oligocene age, which belong to the Corbi Formation [9,15]. These sandstones are characterized by a polymictic composition that includes quartz, alkali feldspar and plagioclase, muscovite granoclasts and metamorphic rock fragments such as gneiss, schists and quartzites [16].

Due to the mineralogy of the grains and the composition of the binder, the Corbi sandstone petrotype is a particular one. The average grain size and the proportion of 40–60% unclogged pores from the total volume of pores determine the permeability, the accumulation of water in the bedrock and the gravitational circulation of water [9]. The research carried out by the team led by Prof. Marin Şeclǎman established a percentage of the water stored in the inner walls of the church of 11%, which would correspond to a maximum porosity of 25%, a value much higher than the real porosity. Water from the Pârâul Cascadei/Cascade Stream brings most of the moisture. The Pârâul Cascadei originates from the Brǎduleţ formation and drains the rocks with sulfates from the Corbi sandstone roof, infiltrating the walls of the church, with priority in the northern one. The water has a flow with seasonal fluctuations and variable compositions, containing predominantly Ca^2+^ and Mg^2+^ ions, chlorides and SO_4_^2−^ ions, which participate in the degradation process, with the formation of gypsum, CaSO_4_·2H_2_O.

The chemical composition established for the wall tiles inside the church also has a high content of uranium (U) and thorium (Th), values that determine a higher natural radioactivity inside the church compared to the radioactivity of the natural background outside it [17]. Measuring the indoor and outdoor dose rate levels and comparing them will express the extent to which the interior is isolated from the exterior [18]. It is possible that radon (with radioactive isotopes produced both by the descendants of thorium and especially by uranium) accumulates inside in higher concentrations than outside. This radioelement is also soluble in infiltration waters, respectively, in the humidity of the air inside the church [19]. In the first constructive forms of the place of worship, when it was insulated from the outside (preserving the walls better) much better than it is today, the radioactivity level of the air inside was most likely much higher. Understanding the risks associated with radon exposure (beyond the states of dizziness, exaltation and other forms that could induce religious trances in the past) and mitigating radon levels through the measures taken will significantly reduce the risk of lung cancer, for example, for those who regularly attend church nowadays [20,21]. Currently, there are kits on the market for testing radon in closed spaces that are simple and relatively cheap. However, the technical solutions for stabilizing the hill and reducing infiltrations should also take into account the long-term minimization of indoor dose rates, with the simplest methods being adequate ventilation but also maintaining a temperature regime specific to the day/night cycles and the succession of seasons. Once the cave complex is stabilized both externally and internally, it is possible to move on to a project to restore the inner walls, including the painting [10].

## 3. Preliminary Dating Characteristics and Radiocarbon Dating

### Materials and Characterization Techniques

The dating of the frescoes can be done from the point of view of identifying the materials and execution techniques used and through the prism of art history but also through physico-chemical analyses to establish their composition (pictorial support, pigments and materials necessary for the creation of frescoes, specific to historical periods).

Intrinsic information can be provided by radiocarbon dating. The result will complement both the conclusions of specialized studies and the investigations that usually accompany them. Thus, compared to the older studies [9,10,11,12,13] or the recent experiences in the field of the RoAMS laboratory from IFIN-HH [22,23], this method provides an answer regarding the age of the investigated materials by means of absolute chronology, in tandem with a series of methods that usually are not associated with radiocarbon dating. This has its limitations, in addition to its advantages [24]. Therefore, it is not important to obtain a radiocarbon date but to interpret it through the prism of all the information held (historical, archaeological, architectural, from the records of the interventions, from the history of art and from the preceding physico-chemical investigations). Based on this preliminary information, the relevant sampling areas can be established from where to extract representative fragments that may contain carbon compounds. Since radiocarbon dating is an invasive technique, the preliminary studies must provide sufficient information to take into account the minimum intervention on the walls/frescoes while obtaining maximum information. A representative sample can be taken, through an appropriate technique, for each wall, which will contain differentiated fragments depending on the studied area.

Based on the characterization through previous studies of the four walls of the church, it was determined that the northern and southern walls remain important in the radiocarbon dating of the first forms of painting on the inner walls [6,7].

Based on the existing information, it was possible to establish the position horizontally in front of the altar and vertically in front of the current floor level from where these samples could be taken, as well as the minimum justified number of required samples. Thus, respecting the deontology of the conservation and restoration of historical monuments [25,26], one sample of mortar/plaster was extracted for each sampling point on each of the two walls, and they were then sub-divided into several fragments, depending on the preliminary results regarding the characterization of the state of preservation of the respective sample, obtained on the remains left after preparing the samples for dating.

**Figure 3:**
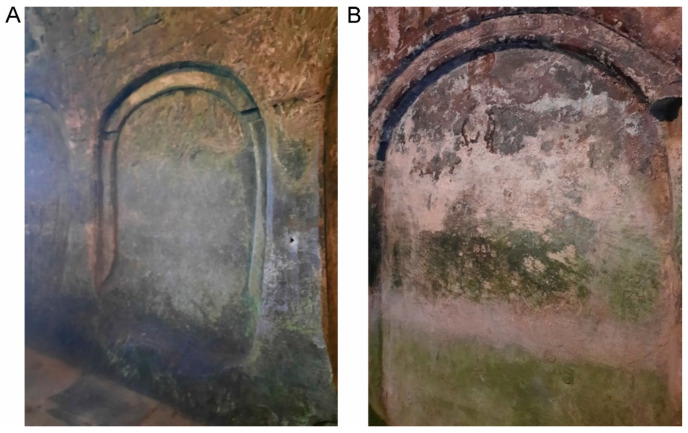
Corbii de Piatrǎ church: (**A**) the second square from the altar on the northern wall from where sample N was taken, divided into fragments N1, N2, N3 for radiocarbon dating (Photo: Simion, C. A); (**B**) the second square from the altar on the southern wall from where sample S was taken, divided into fragments S1, S2 for radiocarbon dating (Photo: Simion, C. A).

The fragments thus obtained for dating, two for the southern wall and three for the northern wall, corresponding to the two chosen areas, were taken from the height of 1.70 m above the floor. The sample on the northern wall was taken from the second square from the altar (Figure 3A), and the one on the southern wall from the corresponding opposite square (Figure 3B).

**Figure 4:**
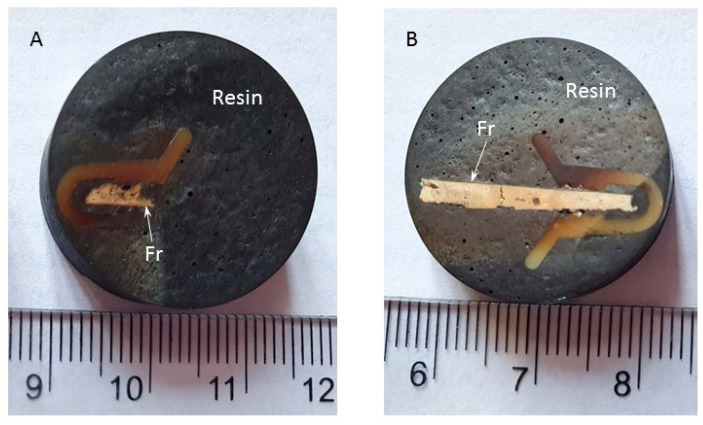
Corbii de Piatrǎ church; details of the leftovers from the samples prepared for radiocarbon dating, used in the preliminary analyses: (**A**) remains from the sample of the northern wall; (**B**) remains from the sample of the southern wall.

The remains were prepared for other physico-chemical investigations necessary for the preliminary characterization. The details of these latter fragments are captured in Figure 4A,B.

According to traditional receipts, all mortars may contain carbon residues from the straw inserts used in the preparation [27]. S-type fragments, taken from the southern wall, are very poorly adherent. The N-type fragments, taken from the northern wall, are very adherent. In addition to the S-type fragments, the N-type fragments could contain sheep tallow or mineral oil (Table 1).

The sub-samples taken from N-type and S-type remains (Figure 4A,B), one for each side, were investigated before the radiocarbon dating through a series of techniques to reveal the initial pictorial composition/at the intervention in direct context with the plaster that will be dated but also information on the materials with which this plaster came into contact over time, whether it is about the lithic support, whether it is about the fresco itself or about its interaction with the environment inside the church. Thus, a direct connection can be made between the age of the pictorial support and the painting in the respective area, but we can also anticipate contaminants containing exogenous carbon that can affect the radiocarbon date of the calcium carbonate, formed by strengthening the pictorial support after being applied to the walls. The characterization of the fresco fragments was carried out by optical microscopy [28,29], cathodoluminescence [30,31,32], scanning electron microscopy with energy-dispersive fluorescence spectroscopy mode for elemental chemical analysis [28,29,33], X-ray diffraction [34,35] and X-ray fluorescence [36,37].

The optical microscopy (OM) characterizations were performed in polarized light with a Zeiss Observer A1m optical microscope, which allows magnification up to 1000×, visualization in bright field, dark field, polarized light and the acquisition of images with a Canon camera Zeiss AG, Oberkochen, Germany.

The microscopic analysis with cathodoluminescence (CL) was performed with Nikon E400, (Nikon company, Tokio, Japan) an optical microscope equipped with a cathodoluminescence device with a cold cathode (CL 8200 MK 3A). The images were acquired with a C.OOLPIX 950 digital photomicrograph device (Keyence International, Mechelen, Belgium). The parameters required to perform the technique were the average vacuum value of 0.5 Torr, current voltage on the bundle of 15–17 kV and current intensity on the electron gun of 350–400 mA, according to the standard in use [30,31]. The interpretation of cathodoluminescence results is qualitative.

The characterization of the fresco element by scanning electron microscopy with energy-dispersive fluorescence spectroscopy (SEM-EDS) was carried out using the HITACHI SU5000 electron microscope equipped with a backscattered electron detector and the energy-dispersive fluorescence spectroscopy module for elemental analysis (Hitachi Groupe, Chiyoda, Tokyo, Japan).

The X-ray diffraction analysis (XRD) is used to complete the identification of the crystalline phases of the mortar components and the painting layer. X-ray diffractions (XRD) were performed using an XPERT PRO MPD 3060 facility from Panalytical (Almelo, The Netherlands), with a Cu X-ray tube (Kα = 0.154051 nm), a 2 Theta of 20°–70°, a step size of 0.13°, a time/step of 51 s and a scan speed of 0.065651°/s. The phase analysis, qualitative, was carried out using the PDXL2 program (Rigaku Corporation, Chiyoda City, Tokyo, Japan) and the PDF4+ 2022 database (International Center for Diffraction Data).

X-ray fluorescence (XRF): the XRF analysis was performed using a portable Bruker Tracer S1 Titan spectrometer (Billerica, MA, USA). A Rhodium (Rh) anode was used to generate an incident X-ray beam to probe the samples. For safety reasons, the energy used is limited to 50 keV. The incident beam is passed through a collimator, resulting in a circular 8 mm in diameter spot on the sample. The resolution for the medium energy domain (Sc to Zr) is approximately 120 eV, according to the manufacturer. Each sample was analyzed in significant zones. The generated spectra were later analyzed using the Bruker Instrument Tools software 1.7.0.128, supplied by the manufacturer.

Additionally, regardless of the chemical form in which it was present in the samples, the following equipment was used for the identification of total carbon (TC), inorganic carbon (IC) and total organic carbon (TOC): PrimacsMCS in combination with FormacsSERIES and the elemental analyzer EA (VarioMicroCube, Elementar, Hanau, Germany)™.

PrimacsMCS allows us to analyze the total carbon (TC) and inorganic carbon (IC) separately. The total carbon is determined by catalytic oxidation of the sample at 1100 °C, while inorganic carbon is determined by digestion of the sample with phosphoric acid H_3_PO_4_ in the IC reactor. Carbon dioxide CO_2_ is detected by the nondispersive infrared NDIR sensor of the FormacsSERIES analyzer. The TOC concentration of the sample is obtained by subtracting TC − IC = TOC.

The radiocarbon dates were obtained at RoAMS laboratory in IFIN-HH using the 1 MV HVEE Tandetron™ system (High Voltage Engineering Europa B.V., Amersfoort, The Netherlands). Before AMS analysis, the samples were converted into graphite by combustion using the AGE 3 graphitization installation [38], which works in conjunction with the elemental analyzer (VarioMicroCube, Elementar, Hanau, Germany)™. The experimental data obtained for all the samples were normalized against NIST SRM 4990C—Oxalic Acid II (NIST SRM 4990C International Standard Reference Material for Contemporary Carbon-14, 1983) to the modern radiocarbon level, while to estimate the blank level, a fossil coal of Romanian origin was used. By measuring the 13C/12C ratio, the results were also corrected by δ13C parameters determined by AMS data processing software version 4.06 (according to A-4-35-501-7621 Rev. A Operator Manual 1.0 MV Tandetron for AMS B7621 IFIN-HH Magurele, Romania, page 73), representing the cumulative isotopic fractionation of all physico-chemical processes on the analysis chain. The ages were calculated using the BATS software tool (Bats version 4.06 (04.06.2015)) [39], according to Stuiver and Polach (1977) [40]. The dating strategy, after characterization of the pictorial layer, should include the determination of the nature of the material and of contaminants, the limitations of methods on the type of material and the analysis of current literature data, whether they are about the type of material to be radiocarbon dated or whether they are about the most appropriate working methodology.

## 4. Experimental Results

**Figure 5:**
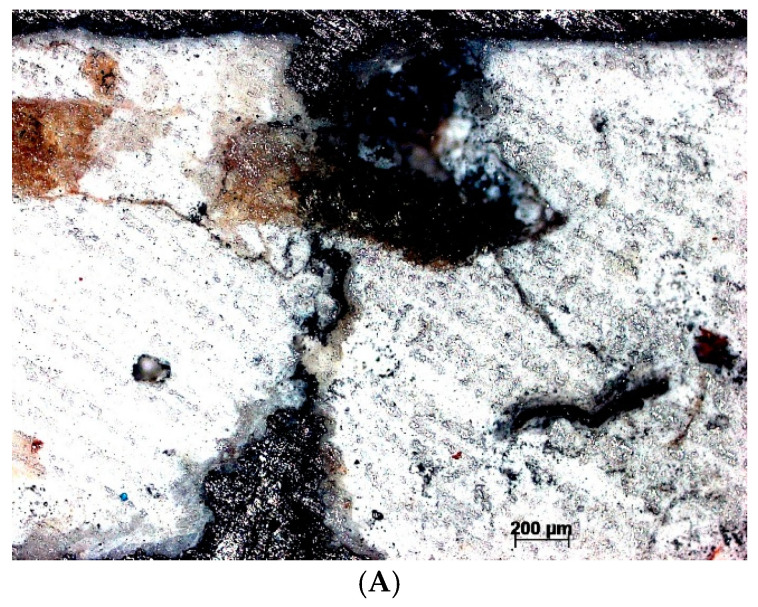

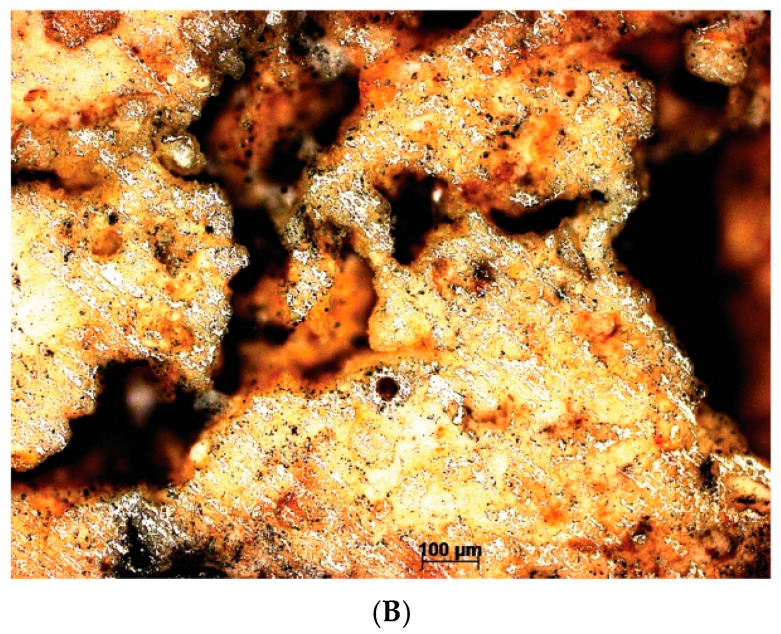
The microstructure of the mortars at the optical microscopic analysis: (**A**) the friable mortar with low adhesion; (**B**) the adhesive mortar with high hardness.

The microscopic analysis in section of the samples highlighted the differences in the microstructure of the two types of mortars: the friable, slightly adherent mortar has a flat surface with traces of the roughness of the abrasive paper (Figure 5A), which reveals the low hardness, while the adherent mortar presents a raised surface that highlights the particles in the mortar that confer a higher hardness (Figure 5B).

**Figure 6:**
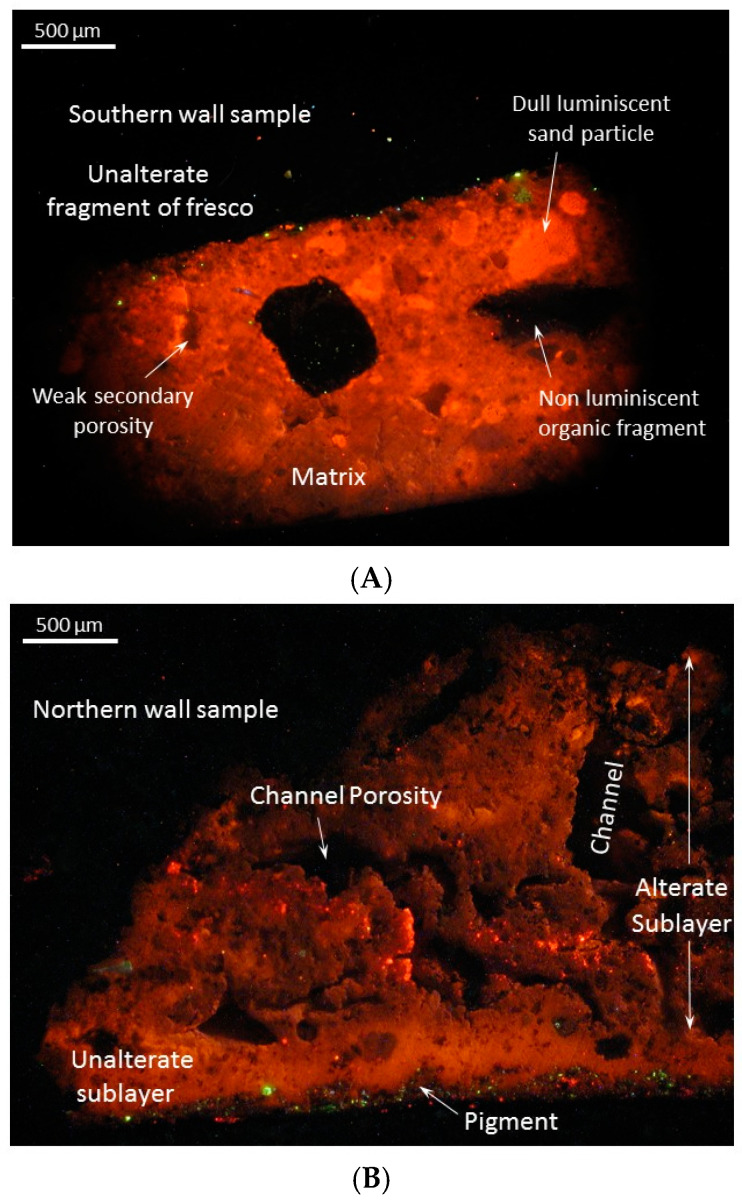
Fragments of fresco under cathodoluminescence microscopy: (**A**) fresco fragment with low adhesion to the sandstone wall; (**B**) fresco fragment with high adhesion to the wall with two distinct areas: an external unaltered sublayer and an internally altered sublayer with high porosity.

The analysis by cathodoluminescence microscopy of the sample with friable mortar highlighted the degradation process over the entire thickness of the layer and the presence of numerous cracks (Figure 6A).

Adherent mortar presents an unaltered outer layer, which includes the pictorial layer with an unaltered internal substrate. Following the massive dissolutions, vacuolar-type secondary pores were formed in canaliform places (Figure 6B). The pores are large, irregular and interconnected, allowing the circulation of interstitial fluids (infiltration waters). This structure suggests the operation of a settling mortar used since ancient times for walls with infiltrations [41]. 

Microscopic analysis by cathodoluminescence highlights two fresco fragments with distinct characteristics. The first fragment of the fresco, taken from the southern wall, is made of a carbonate cohesive matrix with weak orange luminescence, which includes sand particles with various grain sizes from 62 to 1000 µm (Figure 6A). The grains embedded in the matrix are mainly represented by carbonate rock fragments and subordinately by muscovite, feldspar and non-carbonate rock fragments. While carbonates have strong or dull orange luminescence, the feldspars show bright green luminescence and the biotite dull blue-violet luminescence. Nonluminescent fragments of carbonized plants and non-carbonate lithoclasts are also distinguished. An incipient secondary porosity is observed represented by small and irregular pores in low concentration.

The second fragment of the fresco was sampled from the northern wall. This has a more complicated structure, being made up of an unaltered outer sublayer and an altered inner one (Figure 6B). The dominant material of fresco is carbonate, with orange colors and variable luminescence, from bright to dull. The unaltered outer sublayer is compact and has pigment infiltrations on the surface that are signaled by bright vivid colors. The altered sublayer contains an active system of interconnected microchannels (channel porosity), formed in time by selective dissolution processes of carbonate bioclast fragments. The dissolution is a continuous process due to the meteoric waters that percolate the monastery wall [32].

**Figure 7:**
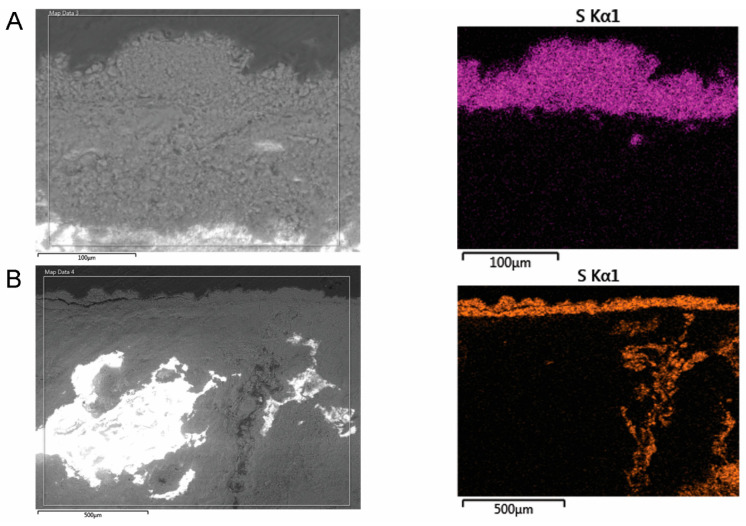
SEM-EDS of the sample with small adhesion in cross-section and elemental mapping: (**A**) surface layer; (**B**) an area where the mortar shows advanced degradation inside.

Characterization by scanning electron microscopy in the section of the mortar sample [42] with low adhesion revealed a degraded structure, with a granular appearance, for which the stratification in relation to the surface of the fresco cannot be established (Figure 7A). In the areas strongly affected by the infiltration water inside the mortar, the EDS mapping analysis highlighted the presence of sulfur from meteoric water (Figure 7B).

**Figure 8:**
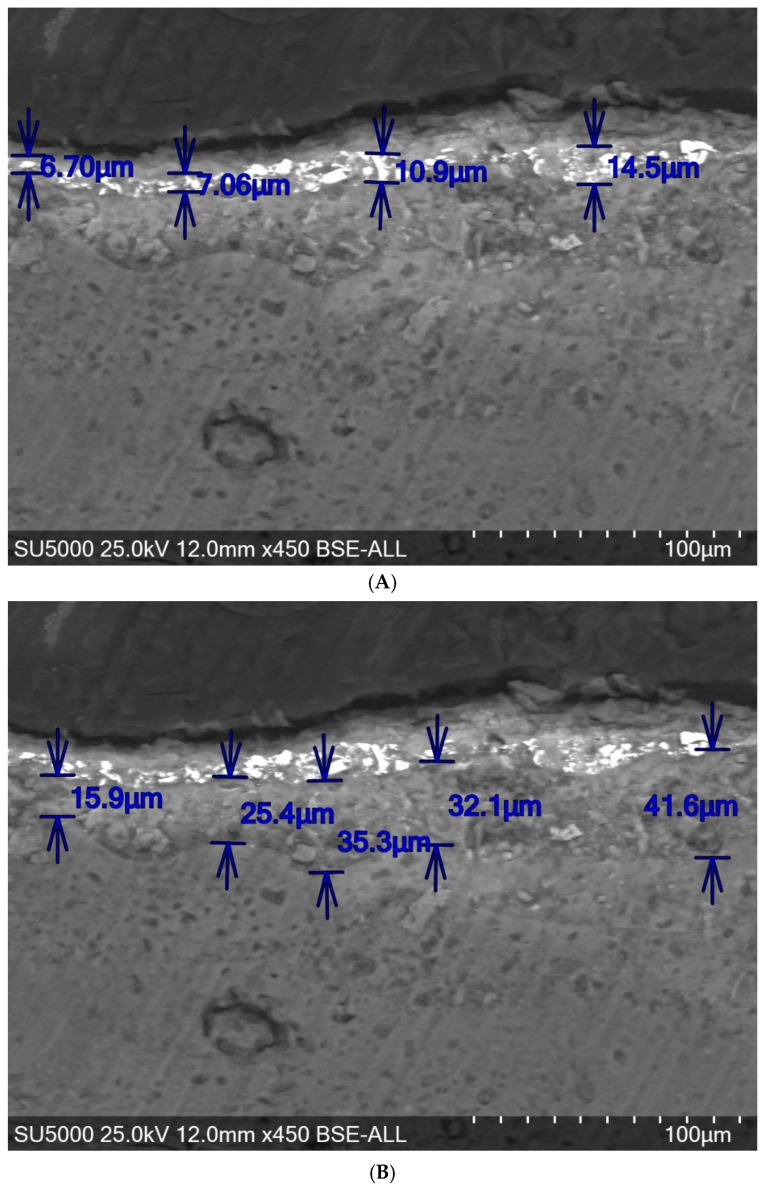
The microstructural analysis in cross-section by scanning electron microscopy of the sample with high adhesion revealed the fresco stratigraphy: (**A**) pictorial layer ×450 WD-10 mm 25 kVcu30si (BSE); (**B**) intonaco layer ×450 WD-10 mm 25 kVcu30si (BSE) [6].

In the case of the adhesive mortar sample, the scanning electron microscopy analysis in section shows that the fresco has kept the initial layering, and the component layers of the fresco have been highlighted as follows: the superficial carbonation layer, the pictorial layer, the intonaco layer and the support layer (Figure 8).

**Figure 9:**
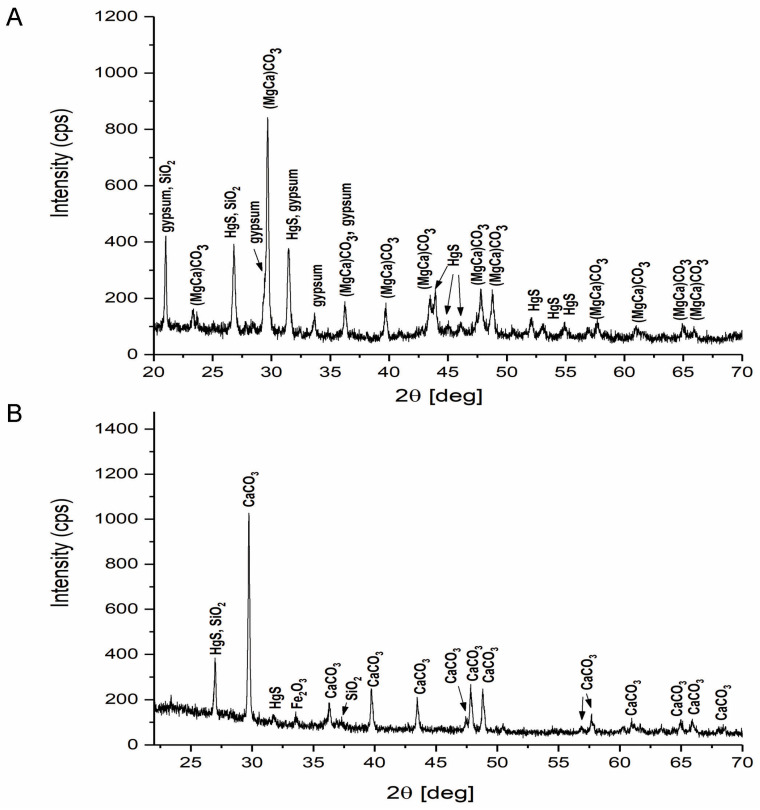
Qualitative phase analysis by X-ray diffraction of pictorial layer of the fresco element: (**A**) with low adhesion to the sandstone wall [7], (**B**) with high adhesion to the sandstone wall [6].

The qualitative X-ray diffraction analysis of the two fresco fragments [42] highlighted the existing phases in the two mortars and in the pictorial layers. The advanced degradation of the fresco fragment with low adhesion is revealed by the presence of gypsum in the friable superficial layer (Figure 9A) as a result of contact with infiltration waters and diffusion processes towards the surface of the fresco.

The high durability and adhesion of the fresco element taken from the northern wall (Figure 9B), the most exposed to the action of infiltration waters, in relation to other fresco areas in the Corbii de Piatrǎ church, is explained by the different structure and properties, especially by the way of degradation with the formation of infiltration water drainage channels. The differences can be explained by the realization of this part of the fresco with a different mortar, in a different period of time than the one in which the full painting of the fresco in the church was done, most likely represented by the samples on the southern wall.

X-ray fluorescence: Compared to previous studies that carried out in situ mapping through dozens of measurement points of the entire surface of the walls and ceiling [10], the current measurements were only performed on the samples that have been taken (Table 2, Table 3 and Table 4). According to the investigations already published, the previously identifiable elements were Fe, K, Al, Mg, Si, Ca, Hg, S, Pb and Mn.

This time, 22 elements were identified not only on the pigment traces but also in the mortar/plaster.

The available information [10,42,43] from XRF prior to the current analysis can be summarized as follows and will implicitly help to select the radiocarbon dating strategy:

- Identified pigments: lead minium, Pb_3_O_4_, applied with the initial addition of an organic binder or during previous interventions (red pigment); white lime; cinnabar (HgS); lead carbonate, PbCO_3_ as a result of degradation or impurity from the lead mine; red and yellow based on iron oxides and coal black;
- Bluish hue: possible mixing of lime white with coal black;
- Possibly azure or malachite in some paintings; possibly an introduction from the time of painting the pediment, like lead white;
- Scheele green pigment CuHAsO_3_ and Schweinfurt green pigment Cu(CH_3_COO)_2_Cu(AsO_2_)_2_, used from the 18th–19th century until the beginning of the 20th century;
- Sulfates and nitrates as salts formed by degradation;
- Alteration products: calcium oxalate; lead carbonate from the alteration of lead minium;
- Analyzes by pigment classes are extensively commented;
- Natural and artificial pigments were used;
- Natural pigments used for ocher and red colors: hematite, limonite, cinnabar;
- Artificial pigments used in white, black, gray and partially red colors;
- Some ingredients are native: limestone, coal, hematite, limonite;
- Cinnabar is “imported”.

The XRF results obtained at this stage highlighted, by applying the Principal Component Analysis (PCA) function of the Minitab 17.0 Program, to what extent the five fragments from the two samples are correlated with each other (testing also the degree of homogeneity/the quality of the partition selection of each sample in several fragments) from the same wall and between different walls. If all the elements determined by XRF are used (Table 2, Table 3 and Table 4), the Score Plot shows a similarity between fragments N2 and N3, respectively, between N1 and S2, with fragment S1 being considered an outlier. The general degree of correlation between all fragments is 92%; it can be an indication of the state of the material from formation to analysis. If only the majority elements are used, and fragments N1, N2 and N3 are separated from S1 and S2, with the general degree of correlation being 86%, it can be an indication of the same type of material but obtained by different recipes. Through the minority elements, N2 and N3 become distinct from S1 and S2, but there is a correlation between S2 and N1; the general degree of correlation is 69%; it shows sources of possibly different raw materials but also the degree of damage by impurities introduced over time from infiltrations from contact with the pictorial material and the surrounding environment. Finally, through trace elements, the following distribution is obtained: N1, N2, N3 and S1 are separated from S2, with the general degree of correlation being 77%.

The interpretation of these results, as well as the information useful for radiocarbon dating, will be presented in the paragraph regarding this method, as in the case of the other analyses.

TC/IC/TOC analysis: The results are shown in Table 5.

Being in small quantity, fragment N1 could not be analyzed. The results are expressed as mass percentages.

The association is observed again on each side of the church; those on the northern side contain more inorganic carbon, most likely carbonates, compared to those on the southern side; a correlation may appear with the degree of friability. The very low percentages of total organic carbon do not exclude deposits due to biological attacks, but they show, a fact also demonstrated in [10], that the straws were not preserved; in their place, small degradation products appeared that will not be able to be separated from such quantities of small fragments/samples so that they can be dated separately. If this isolation/purification had been possible, the radiocarbon age could have fixed the moment of application of the material on the wall much better in time, with the straw being a short-life datable material (annual cycle).

Elemental Analysis (EA): This was carried out practically during the graphitization of the samples for subsequent measurement by AMS. The results are presented in Table 6.

The differences can be noticed between the fragments belonging to sample S on the southern side and those on the northern side, N. The total carbon values are somewhat higher compared to the alternative TC/IC/TOC method (Table 5); this is most likely due to the fact that in both methods, the values are in mass percentages and the base value against which the reporting is done in each case makes it count. The average value of the total carbon during graphitization is approx. 10%; then, the actual carbon contained in the samples subjected to graphitization will enter the range below 35 mg, recommended for the initial mass. It will lead to reduced carbon with a mass within the range [1.7–3.4 mg], acceptable for the chance to obtain approx. 1.52 mg of carbon deposited on the iron catalyst, which is the basis for the AMS target. Nitrogen values, a sign of possible organic compounds, are within the limits of the background of the equipment, so they are most likely not present in the dated samples, being another indication of the absence of degraded organic materials, coming from straw (humic, in general, with significant content in nitrogen).

Radiocarbon dating: The work strategy and the primary interpretation of the results, without placing them in a historical and archaeological context, were based on the information provided by the previous methods. They can be summarized as follows:

Imaging: In regard to differences in structure between the sample on the northern side compared to the one on the southern side, the one on the northern side represents a more compact, adherent material, with short cracks and traces of a possible fatty organic additive (this confirms the hypothesis of its use but will not affect the radiocarbon date, being practically of the same age as when the mortar/plaster on the walls hardened); the one on the southern side is friable and shows cracks propagated over long distances (it may be an indication of the introduction of exogenous carbon of a different age than that in the mortar recipe, in support of this hypothesis comes the identification of sulfur/gypsum formed by infiltrations and deposited in the mortar structure). In the friable fresco, we cannot speak of stratification, and therefore, the better-preserved portions cannot be accurately separated (it was still possible to separate two fragments, S1 and S2, based on the observations during the preliminary analyses), while in the adherent fresco, it is possible to identify all layers in their evolution since formation, which can be separated given the degree of preservation (appreciated implicitly by the lack of sulfur/gypsum deposits) as N1, N2 and N3.

Cathodoluminescence: This highlights the degradative processes in the friable mortar and, therefore, a possible appearance of secondary carbonates of different radiocarbon ages; the adherent one presents an unaltered external layer and an altered internal one with interstitial channels through which the water from the infiltrations circulates freely but preserves the initial signature of carbon-14 in this decanting mortar.

EDS: This highlights the presence of sulfur on the southern side; it becomes a poison for the conditioned iron catalyst in the AGE 3 unit in the case of fragments S1 and S2; that is why it is recommended to use thermal combustion at EA for all five sample fragments, with the subsequent separation of the higher sulfur oxides in the hatch dedicated to their retention.

XRD: This indicates the formation of sulfur-containing gypsum in the fragments on the southern side and shows the differences in the recipe and that the mortar on the northern wall in the investigated area appeared after the initial painting of the church, represented by the selected portion on the southern wall.

XRF: The analyzed samples are basically lime mortar plasters, with traces of pigment. There is a difference between the sample on the southern wall and the one on the northern wall. The fragments on the southern wall probably contain lead minium or lead carbonate formed by its carbonation over time, along with traces of green pigments and/or lead arsenate, a biocide. An argument in support of this last hypothesis comes from the very large amount of arsenic, identified in S1, with a higher concentration in these two elements. Compared to the green pigments identified in previous studies [10,11,12,42,43], used from the 18th–19th century and until the beginning of the 20th century, lead arsenate, introduced later and banned after the 70s in agriculture, was intensively used until the middle of the 20th century and was therefore easy to procure without restrictions on applications, remaining impregnated in the portions of the walls where it was used, which helped to slow down the biocidal attack and preserve the original paintings.

TC/IC/TOC analysis: This confirms the XRF information regarding the lime mortar support, and therefore the possibility of radiocarbon dating. The obtained values show a difference between the north and south sides, which could be revealed by different radiocarbon data. It confirms the practical absence of preserved straw; given the actual situation and the very small quantities of the samples, it will not be possible to separate and purify the inorganic and organic fractions to be dated separately. According to the information from [10], the samples contain calcite and quartz, with mineral impurities originating from the raw materials, from mineral pigments or as a result of aging processes—these will not disturb the radiocarbon dating. The very small amounts of organic carbon identified in the sample on the northern wall come either from traces of straw or from contaminants (possibly sheep tallow or mineral oils for the pictorial support, or as a result of biological degradation); the remains of straw and sheep’s wool would have carbon-14 levels close to those of hardened lime and therefore will not disturb the dating; mineral oil, if present, will lead to much too old data, and biological contamination could lead to much too young radiocarbon data.

The experience of the RoAMS laboratory at IFIN-HH established that, due to the nature of the samples and the very small quantities, it is practically impossible to separate and purify the inorganic and organic fractions to be dated separately. Moreover, the extraction and purification of calcite, even if it is the majority in the samples, could become a risk factor in the sense that it is very possible that in the end, the purified quantities will be too small to meet the conditions for graphitization, especially for N1. Knowing the characteristics of the organic part, there are two risks: one or more radiocarbon ages may be too old (mineral oil, geological carbon from pigment or contamination by infiltration with geological carbonates) or too young (biological attack or exchange with atmospheric carbon dioxide). According to previous studies [9,10,11], it is unlikely that a slow exchange with carbonates from infiltrations or with atmospheric carbon dioxide will occur in the sample from the northern wall, and it is more likely in the sample from the southern wall. Thus, taking into account the risks assumed for objective reasons, the samples were graphitized in raw form in order to measure AMS. The thermal combustion, based on the differential thermal analysis [10], which provided in the diagram a single peak characteristic of calcium carbonate and insignificant peaks for other carbonates or organic compounds, was performed at the elemental analyzer coupled with the graphitization unit. Thus, another set of values was obtained in parallel for the total carbon in the samples, commented on above.

The remaining fragments, after TC/IC/TOC analysis, were finely ground and taken in the quantities indicated in Table 6. The quantity is directly proportional to the carbon/carbonate content established by the other methods. These were subjected to thermal combustion in EA, with the separation, purification and drying of carbon dioxide prior to its introduction into the graphitization unit. The graphitization process, the formation of carbon-containing targets formed by hydrogen reduction on the iron catalyst, the AMS measurements, the calculation of the Conventional Radiocarbon Age (CRA) and the transition from this value to the calibrated age have been extensively exposed in the previous works of RoAMS [44,45,46]. After all these stages, the calibrated ages are obtained, represented by the probability intervals that the historical age (the age of the material, the mortar in this case) is included in it or, with certain statistical weights, in sub-intervals.

**Figure 10:**
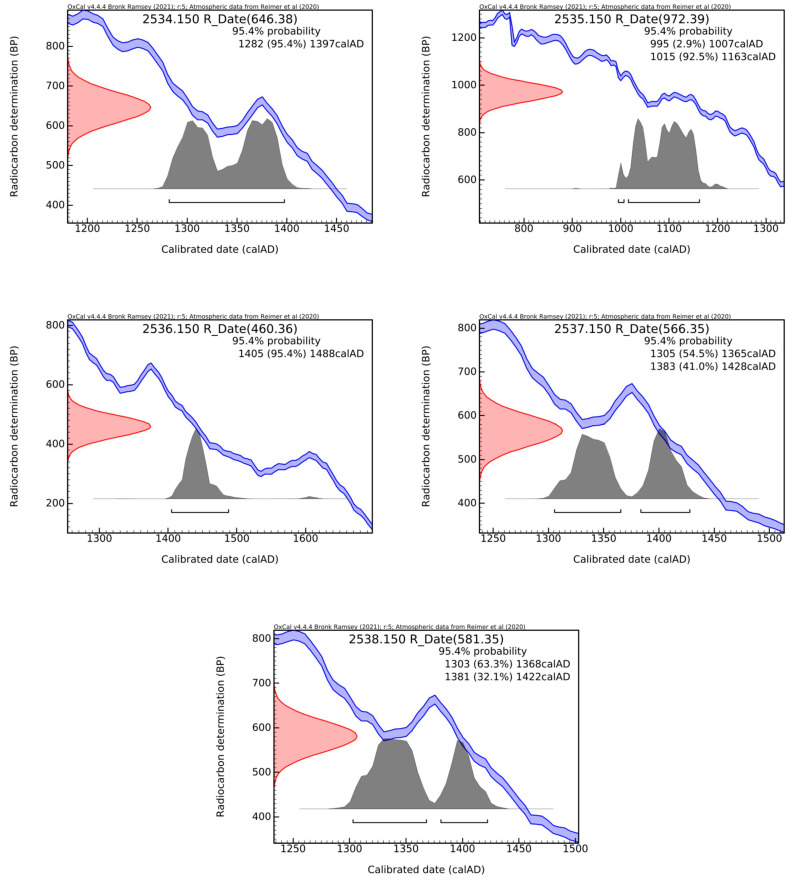
The histograms obtained for the fragments of mortar/lime plasters from the Corbii de Piatrǎ church, separated from the two samples taken, one each for the southern and northern wall, respectively; at the top of each graph, the unique code of the graphitization in RoAMS is indicated (2534.150 for S1, 2535.150 for S2, 2536.150 for N1, 2537.150 for N2, and 2538.150 for N3), as well as the CRA value with the associated measurement uncertainty, both in years BP (according to OxCal version 4.4.4 and [47,48]).

The obtained histograms are represented in Figure 10. The interpretation of the results is done in the next chapter.

## 5. Determination of the Chronology

Determining the chronology involves establishing the contact chronology, through which the relative and absolute dating converge towards a consensus.

Preliminary historical-architectural and archaeological information [1,2,3,4,5,9,10,11]:

15 April, 1456—the oldest documentary attestation through a charter issued by the royal chancellery of the Voivode of Wallachia, Vladislav II, confirming the master Mogoş’s right to rule over the “Corbii de Piatrǎ”;

Proposed, assimilated or documented founders: Basarab I the Founder (ca. 1310–1352), Neagoe Basarab, Radu de la Afumaţi, Lady Ruxandra and Lady Despina (ca. 1487–1554);

Associations with other religious settlements: cave churches from Cappadocia (7th–12th centuries); the church of St. Procopius from Fasano (11th century); hermitages from Bulgaria (13th–14th centuries); small churches from Macedonia (13th–14th centuries); the dating of the altar (10th–14th centuries);

The cutting of the pilaster from the altar and the formation of an altar table, 1m high, the complete restoration of the west wall, currently made of brick, the porch excavated on the south side of the nave: 19th-century interventions;

It was originally intended for small monastic communities; from the 19th century—the laity church (when the transformations occur at the altar);

Elements of dating through the painting of the church: lacunar, summary, placed in the second half of the 14th century by I. D. Ştefǎnescu [49]; in 1973, he returns in another work [50], saying that “it could be two or three decades older than the paintings of the church of Sf. Nicolae-Domnesc at Curtea de Argeş originally built at the beginning of the 13th century by Seneslau; developed by Basarb I (1310–1352) and then by Neagoe Basarab (1512–1521); similar to the Corbii de Piatrǎ;

Immediately or shortly after the excavation/anthropization of the church space in the bedrock, it was decorated with painting; a small part was preserved in particularly bad conditions: fragments in the apses of the altar (east), on the east tympanum, on the southern half of the vault of the nave, on the southern wall of the nave; in the northern part, on the vault and walls, the painting is practically destroyed;

Painted areas on the south wall of the nave: on the upper part of the walls and on the vault;

Painted areas on the north wall of the nave: a single painting fragment visible in the niche next to the altar;

The Deisis in the upper part of the apse of the altar, similarities with the cave chapels of Asia Minor, Southern Italy (10th–13th centuries);

The style of the original painter: c. 12th–13th centuries;

There are also elements that belong to a more advanced phase: the second half of the 13th century—the first decades of the 14th century;

With some effort, one could distinguish two or three “hands” in the original pictorial style;

Not many comments can be made on the chromatics: everything is dull, smoky and altered by mold nowadays;

Broadly speaking, from painting and iconography: end of the 13th century or the first decades of the 14th century.

Analysis of historical documents: the existence of an older foundation of the 16th century; for the previous centuries, there is no written information; after 1503–1506, documents appear due to the Magdalina Nun; she worshiped the monastery of Neagoe Basarab in 1512 in order to be completely “a church for nuns”; from a monetary donation made in 1517 for the commemoration, the legend of the refectory table, dated at IFIN-HH in 2015 and which attests that the wood was cut no earlier than the second half of the 16th century, most likely somewhere after 1725–1813; after 1518, he became a satellite church of the Argeș Monastery; in 1809, it became a wedding church for Transylvanian emigrants from over the mountains (the table in the refectory actually originates from this period, thus refuting the legend that it was stipulated to be the table where Neagoe Basarab took counsel); 1814—the excavation of the pronaos with the modification of the south side and the reduction of the trapeze; 1819—the addition of the wall temple; 1887—the restoration of the west wall, after its collapse; 1890—the erection of the wooden belfry;

The conclusions of the 2008 archaeological survey: no archaeological materials were found in surveys older than the 16th century.

**Figure 11:**
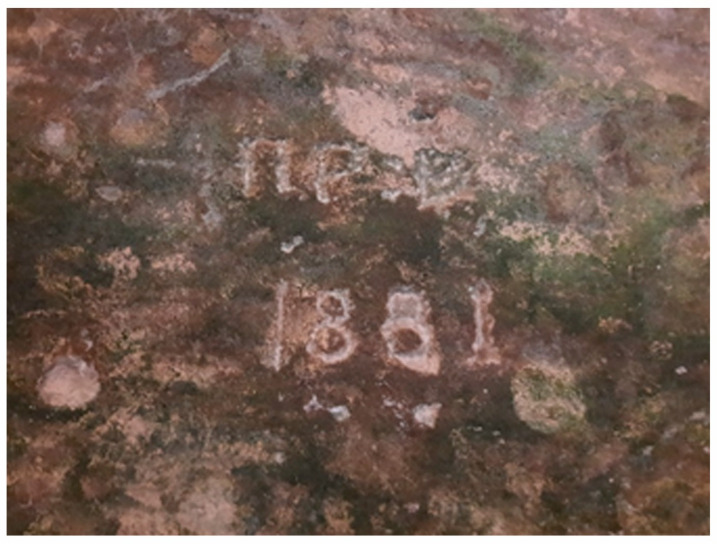
Inscription on the southern wall of the Corbii de Piatrǎ church (Photo: Simion, C. A.).

Inscriptions were visible in 2023, on the southern wall, near sampling point S (Figure 11); with writing specific to the second half of the 19th century, the year 1881 is mentioned, without any reference in the context of the other historical data, with the year being placed before the restoration of the western wall. However, there is also a writing in Slavonic or possibly with elements in Slavonic alternating with elements in Greek, before the transition to the Latin alphabet (ca. 1860), in a register more erased than that of the inscription 1881. In particular, the inscription of the year 1881 does not seem affected by subsequent works, which can advance the hypothesis that after this year, no notable interventions probably took place in the portion of the wall from where the sample from the southern wall was taken.

From the previously published information on the state of conservation of the mural painting, on the support of the mural painting, on the damage to the bedrock caused by meteoric infiltration waters in the inner walls of the church and on the damage to the pictorial support [10], the following conclusions can be drawn:

1. Byzantine painting, with lime-based mortar plaster, applied directly to the bedrock wall:

The painting is almost entirely covered with a biological green layer (the north wall entirely, the south wall up to a level of approx. 2 m of pavement);

Pigments more sensitive to humidity lose their bonds with the binder and form powdery layers when touched;

Decohesion: the migration and recrystallization of salts in many places from the inside to the outside, with the formation of efflorescences and crypto-efflorescences;

The advanced degradation is the result of infiltration of meteoric waters, the natural microclimate, human interventions and the loss over time of the properties of pigments and plaster materials through natural aging;

Meteoric water infiltrations on the northern wall are due to the washing of this wall by the waterfall stream, whose flow rate fluctuates depending on the level of precipitation;

The characterization of two fresco samples with different adhesions, from the northern wall and the southern wall, highlighted the different behavior of the two mortars, with higher adhesion for the northern wall.

2. Different types of mortars, identified as belonging to different centuries/periods of intervention:

Mortars A. from the 14th century, with the preservation of the original painting–partially the walls and the vault of the nave and the altar;

Mortars B. from the 19th century, mortars located on the pediment;

Mortars C. from the 19th–20th centuries, mortars considered for repair, on certain portions of the nave, the lower register of the northern wall, near the arch, from the junction of the door with the southern wall;

Mortars A: lime and straw, porous, biological degradation, repeated recrystallization or induced by freeze-thaw; calcite, traces of quartz, rather impure from the limestone source; sometimes gypsum and feldspar (altar and southern wall) as associated or as mortar degradation products; 75% microcrystalline calcite (from the moment of hardening in the masonry), 3% quartz, 4–5% various minerals; from the differential thermal analysis–the samples contain only calcite due to the binder and do not contain limestone added to the recipe; gypsum–due to the limestone and not to the painting; the straw no longer exists; the original calcite led to phenomena of autogenous healing of the lime, filling in time the gaps in the cracks and pores;

The bedrock in the inner walls is inert and has not interacted with the mortar in the fresco/plaster nor with the pigments in the painting layer; it most likely degraded over time, forming a friable layer at the rock-painting support interface that led to its peeling, a sign of the age of the applied layer; water from the sandstone pores: exogenous soluble salts, dominated by sulfates; appearance of plaster;

The study of the infiltration directions through the bedrock/sandstone shows a predisposition that explains why the northern walls are more exposed than the southern ones;

The appeared efflorescences do not contain calcium carbonates;

The organic component of crusts: lichens;

On the northern wall, the biological colonization is intense, being also identified in the mortar; almost exclusive presence of gypsum on the southern wall;

The specific Liesegang carbonation of lime matured, years before use, with the formation of rings rich in calcite succeeded by bands poorer in calcite, precipitated over time, which finally gives different density to carbonated lime.

An interesting hypothesis that deserves to be studied in the future is the accentuation of the state of friability/degradation due to the combined exposure to radon in the presence of oxygen from the air or from the infiltration waters. It is known that in isolated cavities, such as barrow graves, sudden exposure to air/humidity in the presence of high concentrations of radon accumulated over time induces the friability to the pulverulent state of some perfectly preserved archaeological materials until the time of excavations [51,52].

Interpretation of radiocarbon data:

There are differences between the radiocarbon data of the fragments on the southern side compared to the northern one. Those on the southern side offer older radiocarbon data than the northern one. The results are supported by all the previous analyses presented in the summary before but also by those offered by this study. The dates are included in the 11th–15th centuries AD, in accordance with the presented historical-architectural data.

The result for S2 is slightly older (which would support some hypotheses from the preliminary historical-architectural and archaeological information paragraph), and for N1, it is slightly younger (which would support other frameworks from the same paragraph). Although they could represent application sequences of the pictorial layer, the fragments (N1, N2 and N3 and S1 and S2) are too close to each other to have different ages from different historical contexts. Most likely, some contain contaminants with a different level of carbon-14 that affects the radiocarbon result (as explained by some observations independent of radiocarbon dating). Although they do not vary in the same direction, N1 and S2 have in common this modification of the radiocarbon date from the hardening of the mortar in the masonry and therefore form a distinct group in the PCA analysis of the XRF data.

The results for N2 and N3 correlate perfectly; in both cases, the majority of sub-intervals were placed in the 14th century. This result is supported by the PCA analysis of the XRF data, as in the case of S1. For S1, the estimate would be for the end of the 13th century–the beginning of the 14th century.

The 14th century presents a disadvantage in radiocarbon dating, being considered an “imprecise” century [53]; it can be seen from the histograms in Figure 10 how two split signals of relatively equal intensity are obtained. In the particular historical context of the Corbii de Piatrǎ Ensemble, it will be very difficult to decide, for these samples or for others in the future, if the painting segments belong to the first half of the 14th century, therefore associated with Voievod Basarab I (1310–1352), or if there are interventions from a second half of the 14th century. Anyway, the results exclude the interventions of the 16th century (Neagoe Basarab) and highlight the initial forms of the painting.

If we use the R_Date Combination function in OxCal (https://c14.arch.ox.ac.uk/oxcal.html, accessed on 26 January 2024) for N2, N3 and S1, then we will obtain a radiocarbon date with better precision, ±21 years BP, which passes the χ-Test. It gives a clearer situation for interpretation: the paintings on the two walls represent an early pictorial form currently identifiable with the following statistical weight: for σ = 2, there is a 74.0% probability that the time of execution of the painting is placed in the interval calAD 1305–1365, and for σ = 1 there is a dominant probability of 56.2% that the interval is restricted to calAD 1321–1358. Most probably, these painting segments belong to the first half of the 14th century and, therefore, are associated with Voievod Basarab I (1310–1352). Anyway, the results exclude the interventions of the 16th century (Neagoe Basarab) and highlight the initial forms of the painting.

Finally, I. D. Ştefǎnescu correctly defined in 1973 the correspondence with Sf. Nicolae-Domnesc at Curtea de Argeş church, which was originally built at the beginning of the 13th century by Seneslau and developed by Basarab I (1310–1352) and then by Neagoe Basarab (1512–1521).

## 6. Conclusions

Mortar/plaster samples associated with pictorial layers documented as being from the first forms of painting of the inner walls of the church from the Corbii de Piatrǎ Rupestre Monastery in Romania were analyzed.

During sample collection, it was observed that large sections of the fresco had low adhesion, while small portions were highly adherent to the wall. The specific structural components of the intonaco mortar used include the matrix, hard particles and straw insertions.

The low-adherence, friable mortar sampled from the southern wall is severely degraded, exhibiting large pores and cracks that propagate perpendicularly to the fresco surface due to wetting/drying cycles or developing randomly from the tips of the straw insertions. Areas of advanced degradation contain sulfur carried by rainwater from the slopes, which it washes before infiltrating the church walls. The mineralogical characterization of the mortars is framed within the geological context of the site. From a geological point of view, the site containing Corbii de Piatrǎ Monastery is located in the Getic Depression, a narrow sedimentary basin in the Southern Carpathian foreland with sediments from Cretaceous to Miocene age [14]. The monastery was excavated in massive sandstones of the Oligocene age, which belong to the Corbi Formation [8]. These sandstones are characterized by a polymictic composition that includes quartz, alkali feldspar and plagioclase, muscovite granoclasts and also metamorphic rock fragments such as gneiss, schists and quartzites [9,14,15,16].

Similar studies have been conducted on fresco paintings in Cappadocia from the 9th–12th centuries [54,55,56,57,58] and on frescoes in Orthodox churches in Bulgaria [42].

The adherent mortar sampled from the northern wall, through which water infiltrates, is compact, with few short cracks and separations of a fatty additive, possibly sheep tallow, which was used in that period to enhance the mortar’s properties. In this mortar, rainwater infiltration created a network of large, interconnected pores forming channels that allowed water transfer through the mortar to the middle layer without degrading the masonry–fresco interface or the mortar–pictorial layer interface. This behavior functioned as a settling mortar, which helped the fresco maintain its adhesion to the wall.

To support the radiocarbon dating strategy and the results obtained by this method, a series of physico-chemical investigations were preliminarily carried out on small portions left after the preparation of the two samples for radiocarbon dating (microscopy, elemental analysis, identification of natural compounds or formed as a result of the degradations) with the aim of separating several individual fragments and their separate dating (the consistency of the radiocarbon data originating from the endogenous carbon is monitored).

Techniques were used that cover a wide and complementary palette of analyses, completing the existing studies, and the conclusions are convergent: the fragments represent lime mortars, different in appearance, constitution and behavior.

Regarding the radiocarbon strategy, the results of the preliminary analyses provided the following information: the percentage of calcite in the sample, the content of impurities, the presence/absence of organic materials bearing exogenous carbon (with a carbon-14 level different from that at the time the mortar hardened in the plaster) and the extent to which the last two categories could affect the radiocarbon date.

Thus, there were enough arguments to support direct AMS dating, without pre-treatment. The results for the northern wall are different from those for the southern wall. The latter provide older ages but are not old enough to be a clear separation from the results for the northern wall. In both cases, there are even older and newer ages that could be supported by some historical and architectural data, but the PCA analysis of the XRF results shows that the outliers are most likely a sign of carbon-14 contamination levels different from the one at the time of hardening of the mortar on the wall.

From the point of view of historical information, the data up to now support the idea of two close phases of painting: a first phase revealed on the southern wall, which belonged to a painting during the time of Voivode Basarab I the Founder (1310–1352), and a second phase highlighted on the northern wall that was lacunar, brief and framed in the second half of the 14th century by I. D. Ştefǎnescu.

The fact that the adherent fresco on the northern wall is slightly more recent than the friable one on the southern wall suggests that these are restored areas, following a degradation process, using a mortar with superior properties.

Being extremely degraded, further invasive analyses will no longer be allowed. Therefore, in order to obtain additional information on the areas of the initial painting, a mapping with an XRF/FT-Raman portable instrument [54] can be used, highlighting, compared to previous studies, the traces of the biocide applied to the mural painting 100 years ago. Moreover, with the help of radon determinations, other phenomena can be highlighted that can affect both the painting and the health of the people who come to religious services. This is due to the sandstone from which the bedrock is formed, with the percentages of uranium and thorium from the natural radioactive series being quite significant.

## Figures and Tables

**Table 1:** Fragments from the samples used for radiocarbon dating and their masses.

Sample Name	Weight [mg]
S1	265.7
S2	243.5
N1	39.1
N2	196.5
N3	351.0

**Table 2:** XRF results, without errors, in ppm; all identified elements; brings together types of materials, construction periods/restorations and recipes + sources of raw materials.

Sample Name	Mg	Al	Si	P	S	K	Ca	Ti	V	Cr	Mn
S2	5474	0	165,353	0	2308	0	183,631	135	0	69	197
S1	7543	1055	133,947	2060	55,834	0	162,457	352	33	36	383
N3	7138	7578	277,789	616	1082	1082	128,797	1596	0	92	257
N2	3863	6968	203,358	0	2654	1406	38,027	907	33	15	182
N1	6255	0	137,900	494	2725	0	77,282	122	0	0	136
S2	Fe	Ni	Cu	Zn	As	Sr	Y	Mo	Pb	Th	U
S1	9720	111	33	104	84	197	14	0	19	0	0
N3	24,554	128	0	561	3946	322	74	11	1421	19	176
N2	17,850	43	33	38	0	101	0	0	0	0	0
N1	12,689	127	40	56	0	113	0	0	0	0	0
S2	2498	182	77	75	0	127	0	0	0	0	0

**Table 3:** XRF results in ppm for elements from Mg through Mn, errors included.

Sample Name	Mg	Err	Al	Err	Si	Err	P	Err	S	Err	K	Err	Ca	Err	Ti	Err	V	Err	Cr	Err	Mn	Err
S2	5474	3077	0		165,353	1170	0		2308	131	0		183,631	325	135	22	0		69	10	197	12
S1	7543	3299	1055	618	133,947	1085	2060	191	55,834	346	0		162,457	318	352	26	33	14	36	11	383	16
N3	7138	2955	7578	674	277,789	1472	616	179	1082	110	1082	92	128,797	279	1596	36	0		92	11	257	15
N2	3863	1945	6968	567	203,358	1163	0		2654	90	1406	59	38,027	137	907	24	33	12	15	8	182	10
N1	6255	2106	0		137,900	968	494	120	2725	96	0		77,282	192	122	16	0		0		136	10

**Table 4:** XRF results in ppm from Fe through U, errors included.

Sample Name	Fe	Err	Ni	Err	Cu	Err	Zn	Err	As	Err	Sr	Err	Y	Err	Mo	Err	Pb	Err	Th	Err	U	Err
S2	9720	61	111	22	33	22	104	14	84	15	197	10	14	8			19	6				
S1	24,554	109	128	23			561	26	3946	64	322	12	74	10	11	5	1421	21	19	2	176	16
N3	17,850	76	43	16	33	15	38	9			101	7										
N2	12,689	59	127	22	40	19	56	11			113	8										
N1	2498	30	182	28	77	26	75	14			127	11										

**Table 5:** Total carbon (TC), inorganic carbon (IC) and total organic carbon (TOC) values.

Sample Name	TC [%]	IC [%]	TOC [%]
S1	8.88 ± 0.44	8.56 ± 0.43	0.32 ± 0.01
S2	8.99 ± 0.45	8.45 ± 0.42	0.54 ± 0.03
N2	9.86 ± 0.49	9.86 ± 0.49	Near 0
N3	10.09 ± 0.50	9.55 ± 0.48	0.54 ± 0.03

**Table 6:** Elemental analysis results.

No	Weight [mg]	Sample Name	Lab Code (RoAMS)	N Area	C Area	N [%]	C [%]	C/N Ratio
1	34.31	**S1**	G4896_2534.150	8657	92113	0.67	10.13	15.01
2	16.92	**S2**	G4897_2535.150	8238	45221	1.30	10.08	7.76
3	19.09	**N2**	G4898_2537.150	825	52457	0.12	10.36	88.95
4	19.28	**N3**	G4899_2538.150	894	57986	0.13	11.35	89.81
5	17.21	**N1**	G4900_2536.150	1242	44391	0.27	9.73	49.09

## Data Availability

The original contributions presented in this study are included in the article. Further inquiries can be directed to the corresponding authors.
